# The Relationship Between Multidimensional Poverty and Depression Among Single-Parent Families: A Comparative Analysis by Gender of the Household Head

**DOI:** 10.3390/healthcare14172723

**Published:** 2026-08-26

**Authors:** Jinkyung Park, Donghyeon Kim, Daeyeon Jang

**Affiliations:** 1 School of Social Welfare, Yonsei University, 50 Yonsei-ro, Seodaemun-gu, Seoul 03722, Republic of Korea; pjk77800@gmail.com (J.P.); alskdj2010@yonsei.ac.kr (D.K.); 2 Institute of Social Welfare, Department of Social Welfare Counseling, Hankyong National University, 327, Jungang-ro, Anseong-si 17579, Gyeonggi-do, Republic of Korea

**Keywords:** single-parent families, multidimensional poverty, depression, cumulative risk model, gender differences

## Abstract

**Background/Objectives:** This study examines gender differences in the relationship between multidimensional poverty and depression among single-parent families, drawing on the concepts of the feminization of poverty and dual vulnerability to inform gender-sensitive policy. **Methods:** Using a cross-sectional design, this study analyzed data from the 2024 Korean National Survey on Single-Parent Families, with a sample size of 3315 single-parent families. Multidimensional poverty was assessed across five domains: income, employment, housing, health, and social relations. Depression was measured using the Patient Health Questionnaire-9 (PHQ-9). **Results:** Poverty levels were highest in income, followed by housing, health, social relations, and employment. Female single parents experienced significantly higher poverty in income, employment, and social domains compared to males, while male single parents showed higher asset poverty. The proportion of respondents in the depressed group did not differ significantly by gender, though male and female single parents reached this similar overall level through partly different configurations of risk. Employment, health, and discrimination were common predictors of depression across genders, while lower education was an additional risk factor specific to female single parents. Depression scores rose in a linear, dose-dependent manner with the accumulation of multidimensional poverty for both genders, with a formal test indicating no significant overall difference in this pattern by gender. **Conclusions:** The findings suggest a critical need to shift policy from simple cash transfers to an integrated, multidimensional paradigm encompassing housing, employment, health, and stigma reduction. Policy interventions must prioritize gender-sensitive approaches tailored to the distinct risk configurations identified for male and female single parents.

## 1. Introduction

As family structures in Korea have become increasingly diverse, growing attention has been directed toward the development of policies and support systems for various family types, including large families, single-parent families, grandparent-headed households, and multicultural families. Among these, single-parent families represent one of the most prominent blind spots in the welfare system and are widely recognized as a highly vulnerable population. As of 2024, single-parent households numbered approximately 1.5 million, accounting for 6.5% of all Korean households [1]. Although institutional support has been provided under the Single-Parent Family Support Act, which evolved from the Mother and Child Welfare Act enacted in 1989, single-parent families continue to experience serious structural difficulties. Divorce or separation is the most common pathway to single parenthood, accounting for 84.2% of cases [2], and South Korea now ranks second among Asian OECD countries in divorce rate [3]. Economically, the average monthly income of single-parent families is 2.946 million KRW—only 60.3% of the average household income—reflecting severe financial hardship. Moreover, 65.9% of these households rely on government assistance [2]. Nearly half of employed single-parent household heads earn less than 2 million KRW per month, and 10% earn less than 1 million KRW [4]. Asset holdings, moreover, amount to only about 20% of those of two-parent families, and female-headed households report income levels equivalent to just 70% of male-headed households, indicating a pronounced gender disparity within this population [4]. Despite increased social acceptance of divorce, 16.7% of single-parent families continue to experience discrimination [2].

The vulnerability of single-parent families, however, is not confined to material deprivation but extends into non-material and relational domains such that single-parent families experience poverty in multidimensional ways. Care gaps represent a critical issue, particularly for employed single mothers who often lack access to alternative caregivers or sufficient time to care for their children themselves [5]. Among elementary and secondary school children in single-parent families, 51–53% are reported to be left unattended—a rate six to eighteen times higher than that observed in two-parent households [2]. Single-parent household heads, regardless of gender, also experience excessive role strain resulting from the need to fulfill both parental roles simultaneously [6,7]. In addition, both parents and children frequently encounter discrimination in neighborhoods, workplaces, and schools. Single mothers in particular report significantly higher levels of depression, anxiety, and stress compared to other women, driven by compounded parenting stress, restricted labor market opportunities, and structural disadvantage within a gender-unequal society [8,9]. These accumulated pressures carry measurable psychological consequences: the prevalence of depression among single-parent household heads reaches 20.2% [10], a rate understood not merely as a result of economic deprivation but as an outcome of prolonged marginalization and accumulated stress [11]. Consistent with the Cumulative Risk Model, individual poverty-related risk factors independently influence depression, while overlapping risks intensify depressive symptoms in a compounding, non-additive manner [12,13], suggesting that the deprivation experienced by single-parent families operates through interactive rather than merely additive mechanisms.

This interactive pattern of risk points to a fundamental limitation in how poverty among single-parent families has conventionally been conceptualized: measures confined to income are insufficient to capture the layered and interacting disadvantages this population faces. The concept of multidimensional poverty emerged precisely as a critique of and an alternative to approaches that define poverty solely in economic terms. Rather than reducing poverty to material deprivation defined by income or asset shortages, multidimensional poverty incorporates a broad range of factors that constitute or drive social exclusion in contemporary society, including housing conditions, employment, health, social and cultural participation, and interpersonal relationships [14]. From this perspective, income poverty represents only one component of poverty, and its accurate measurement requires attention to the diverse and heterogeneous dimensions of human welfare needs [15]. This framing is closely aligned with Townsend’s concept of relative deprivation, encompassing not only material resources such as food, clothing, and housing, but also social activities including labor, education, and family relationships, as well as broader lifestyle and social participation [14,16]. This orientation toward welfare as a multidimensional and interrelated phenomenon builds on Sen’s capability approach, which reframes deprivation as the inability to achieve valued functioning rather than as a shortfall in income alone [17], and on the Alkire–Foster counting methodology that operationalizes this idea through a dual-cutoff procedure identifying individuals as multidimensionally poor when they are deprived in a sufficient share of weighted indicators [15], which underpins the global Multidimensional Poverty Index.

Despite the conceptual strength of this framework, poverty among single-parent families has rarely been examined from a multidimensional perspective, and no established measurement criteria specifically suited to this population currently exist. Dimension-specific indicators developed for other groups, such as elderly populations, present clear limitations when applied to single-parent families. Employment measures based on job type or job satisfaction implicitly assume the individual is already employed, failing to capture deprivation associated with unemployment itself. Medical-expenditure-based health indicators fail to identify individuals who cannot access healthcare precisely because of financial constraints. Housing deprivation likewise cannot be adequately captured by homeownership status alone. And family/social dimensions require attention to gender-based differences and experiences of discrimination, rather than treating single-parent status itself as the sole marker of deprivation. These gaps indicate that a tailored multidimensional poverty framework—one that reflects the specific economic, employment, housing, health, and social circumstances of single-parent families—is needed. Accordingly, this study aims to systematically measure multidimensional poverty among single-parent families across five domains—income, employment, housing, health, and social relations—and to examine both the independent and cumulative effects of these factors on depression. Furthermore, this study explores whether the relationship between multidimensional poverty and depression differs by the gender of the household head (single mothers versus single fathers), thereby providing empirical evidence for the development of gender-responsive policy interventions.

## 2. Materials and Methods

### 2.1. Data and Respondents

This study utilized data from the 2024 Survey on Single-Parent Families. Conducted every three years since 2012 in accordance with Article 6 of the Single-Parent Family Support Act, this survey is a state-approved statistical instrument designed to collect baseline data for establishing mid-to-long-term support policies [2]. The target population for the current survey was drawn from single-parent households identified in the 2022 Register-based Census by Statistics Korea. Samples were extracted based on region and household type, resulting in a nationwide total of 3315 single-parent household heads (1118 male and 2197 female) raising children aged 18 or younger. Eligible respondents were, by design, the household head of the sampled single-parent family; the respondent and the single parent under study are therefore the same individual in every case, and no other family member could have served as the respondent. Accordingly, household gender refers to the gender of this respondent, i.e., the single-parent household head.

### 2.2. Measures

#### 2.2.1. Depression

Depression was measured using the Patient Health Questionnaire-9 (PHQ-9), translated into Korean by Park et al. [18]. Originally developed by Spitzer et al. [19], the PHQ-9 has been translated and validated in more than ten countries and is widely used due to its brevity, ease of administration, and effectiveness as a depression screening tool. The PHQ-9 assesses depressive symptoms experienced over the past two weeks using a 4-point Likert scale, ranging from not at all (0) to nearly every day (3). Scores are interpreted as follows: no depression (0–4), mild depression without functional impairment (5–9), moderate depression with functional impairment (10–19), and severe depression requiring clinical intervention (20–27).

#### 2.2.2. Multidimensional Poverty

The independent variable of this study, multidimensional poverty, was defined by establishing poverty lines based on eight specific criteria across five dimensions—income, employment, housing, health, and social relations—following the framework of Kim et al. [14]. The detailed operational definitions for each dimension are as follows.

##### Income Dimension 1: Absolute Poverty

Regarding the income dimension, absolute poverty was applied as the criterion. While absolute poverty generally refers to cases where recognized income is below the minimum cost of living [20], many previous studies have used less than 50% of the median income—the OECD standard—as the poverty line [14]. In South Korea, since the selection of National Basic Livelihood Security (NBLS) recipients is based on these same criteria, status as an NBLS recipient was used as the proxy for absolute poverty. Accordingly, individuals who were recipients at the time of the survey were classified into the poverty group (1), while those who were former recipients or had never been recipients were classified into the non-poverty group (0).

##### Income Dimension 2: Asset Size

While research on the criteria for asset poverty is still ongoing, this study adopted the standards presented in the previous literature [21]. Specifically, based on the 2018 standards, the threshold was set at six months’ worth of 50% of the median income. If an individual’s net assets (total assets minus liabilities) were lower than this threshold, they were classified into the poverty group (1); otherwise, they were classified into the non-poverty group (0).

##### Employment Dimension 1: Employment Status

The poverty line for the employment dimension was established based on employment status. Specifically, those who were unemployed at the time of the survey were classified into the poverty group (1), while those who were employed were classified into the non-poverty group (0).

##### Housing Dimension 1: Housing Area

The standard for the housing dimension was measured by applying the residential floor area. The current minimum housing standards in South Korea have limitations in adequately reflecting the diverse living needs of various household types. To address this, this study adopts the appropriate housing area standards based on the Mankiw–Weil (MW) model proposed by Mankiw and Weil [21]. According to Kim [22], who applied this model to domestic renter households, the recommended standards are 33.0 m^2^ for one-person households, 49.5 m^2^ for two-person households, 59.4 m^2^ for three-person households, 62.7 m^2^ for four-person households, and 66.0 m^2^ for households with five or more members. Based on the survey item ‘What is your current residential floor area?’ from the Survey on Single-Parent Families, households falling below these standards were classified as the poverty group (1), while those meeting or exceeding the standards were classified as the non-poverty group (0).

##### Housing Dimension 2: Housing Type

Regarding housing tenure, households residing under jeonse (a lump-sum deposit lease), monthly rent with a deposit, or monthly rent without a deposit were classified as experiencing housing instability [23].

##### Health Dimension: Health Status

Poverty based on health status was operationalized based on the study by Yoo [24]. In the previous study, health-dimensional poverty was defined as the intersection of two conditions: (1) responding ‘Poor’ or ‘Very Poor’ on a Likert scale for ‘Subjective Health Status,’ and (2) having annual medical expenses exceeding 20% of disposable income. However, such criteria have limitations in excluding individuals who suffer from poor health but cannot afford to visit a hospital due to financial constraints. Therefore, this study did not utilize the medical expense ratio. Instead, individuals were classified into the poverty group (1) if they responded ‘Yes’ to both of the following items: ‘Are your daily activities limited due to health problems?’ and ‘Have you ever been unable to go to the hospital due to financial reasons despite wanting to?’. Otherwise, they were classified into the non-poverty group (0). This indicator shares conceptual content with the depression outcome, as activity limitation is a functional item also captured by the PHQ-9. A sensitivity analysis excluding this indicator confirmed that the study’s core findings do not depend on its inclusion, and it was therefore retained in the primary analysis.

##### Social Dimension: Experiences of Discrimination

Social dimension was defined based on experiences of discrimination to a single-parent and/or their children. Respondents who reported either direct experiences of discrimination or having concealed their single-parent family status were coded as 1, and all other cases were coded as 0.

##### Cumulative Multidimensional Poverty Index

To capture the accumulation of deprivation across domains, a cumulative poverty index was constructed by summing the eight binary poverty indicators described above, yielding a count ranging from 0 to 8. Because the income, housing, and social dimensions each comprise two indicators while employment and health comprise one each, these three dimensions implicitly carry twice the weight of employment and health in the raw count; this reflects the number of indicators, rather than a substantive judgment that these dimensions matter more. For analysis, the raw count was grouped into four categories (0–1, 2–3, 4, 5 or more), with the 4-indicator threshold following the Alkire and Foster [15] convention of treating deprivation in at least half of a household’s indicators as a meaningful cutoff, and the upper category set at 5 or more to avoid unstable estimates given the small number of cases at the highest counts (7 indicators: *n* = 40; 8 indicators: *n* = 6).

#### 2.2.3. Control Variables

Control variables included demographic characteristics that may influence depression: age, educational level, household size, duration of single parenthood, and age of youngest child. Age was measured as the respondent’s age in 2024. Educational level was measured using the respondent’s highest level of education (1 = no formal education, 2 = elementary school, 3 = middle school, 4 = high school, 5 = associate degrees, 6 = bachelor’s degrees, 7 = graduate school or higher). Household size was measured as the total number of household members, including the respondent. The duration of single parenthood was entered as a continuous variable measured in years, and age of youngest child was measured by the school level of the youngest child; the items were categorized as follows: 1 = pre-school, 2 = elementary school, and 3 = middle school and above.

### 2.3. Statistical Methods

First, frequency analysis and descriptive statistics were conducted to examine the general characteristics of the study respondents and the status of multidimensional poverty. To test differences in key variables by the gender of the single-parent household head, chi-square tests were performed. Weighted linear regression was then conducted in two stages to examine the relationship between multidimensional poverty and depression: first, using the eight individual poverty indicators as predictors, and second, using the cumulative poverty index. Both stages were repeated with a gender-by-poverty interaction term included in a pooled model to directly test whether these associations differed by household gender, following a joint Wald test on the interaction terms. All analyses applied the standardized survey weight provided with the dataset; because the released data do not include stratum or cluster identifiers, each observation was treated as an independent sampling unit, a conservative approximation to a full design-based variance estimate. There were no missing values on any study variable. Variance inflation factors for all predictors in the multivariable models ranged from 1.02 to 2.12, indicating no evidence of problematic multicollinearity among the poverty indicators. SPSS 29.0 and Stata 18.0 were used for the analysis.

## 3. Results

### 3.1. Demographics of the Respondents

The demographic characteristics of the respondents are shown in Table 1. The majority of respondents (56.6%) were in their forties. The proportion under 40 differed substantially by gender—13.8% of males compared to 27.8% of females, more than double the average rate (χ^2^ = 196.976, *p* < 0.001). Regarding education level, 55.2% had graduated high school, but among males, 22.2% had a Bachelor’s degree or higher, compared to 20.3% of females (χ^2^ = 21.803, *p* < 0.001). The most common household size was two members, accounting for 45.4%, with males at 40.7% and females at 47.8% (χ^2^ = 17.549, *p* < 0.01). The most common duration of single parenthood was 5–10 years among males (44.2%), whereas among females it was less than 5 years (42.9%) (χ^2^ = 17.522, *p* < 0.001). The proportion with the youngest child in middle school or higher was 51.3%, with males at 55.6% and females at 49.0% (χ^2^ = 20.557, *p* < 0.001). The mean depression score for the total sample was 2.90 (SD = 3.96). Females reported a significantly higher mean level of depression (M = 3.03, SD = 4.06) compared to males (M = 2.66, SD = 3.74) (*t* = −2.61, *p* < 0.01).

### 3.2. Multidimensional Poverty Differences by Household Gender

**Figure 1:**
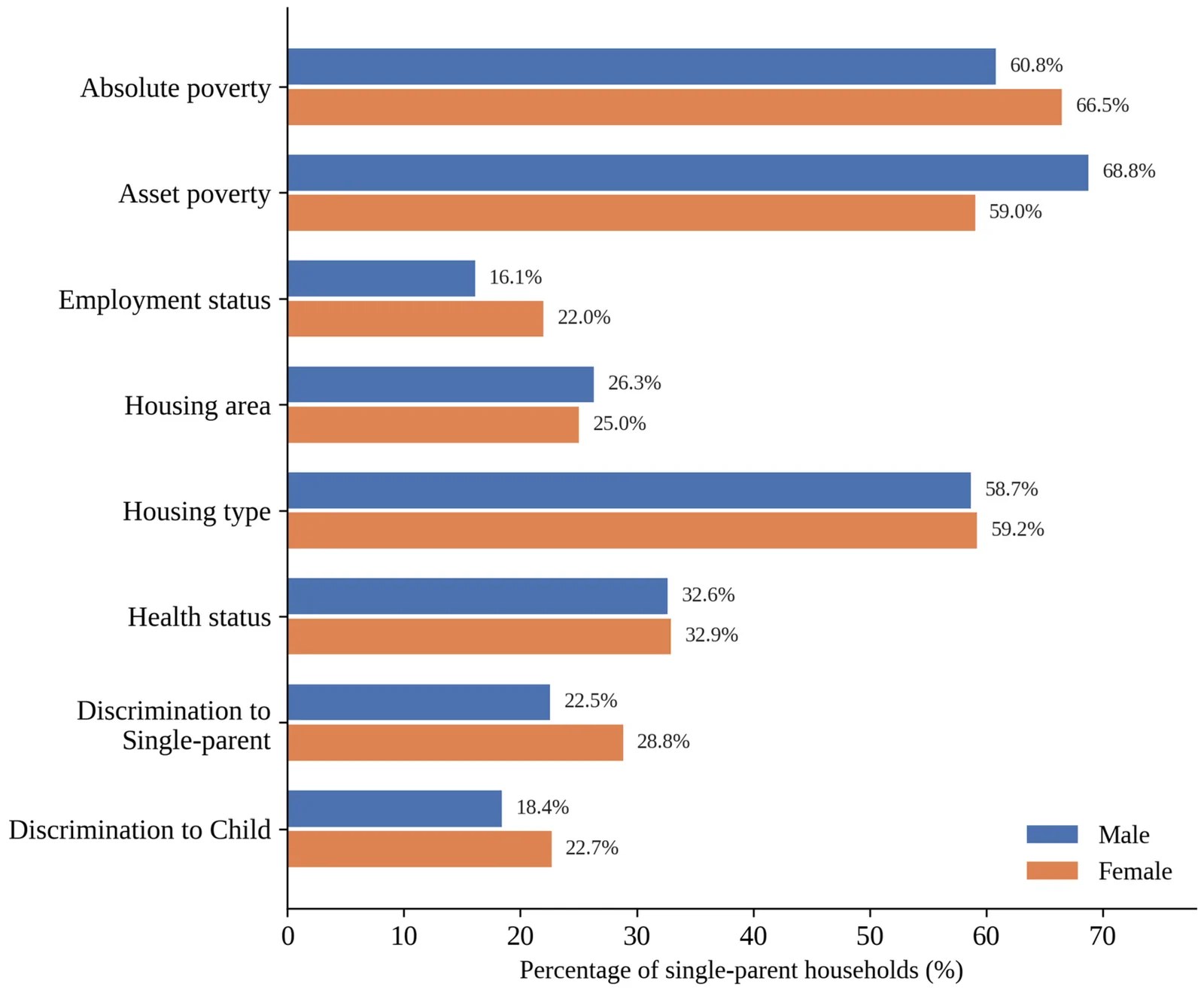
Multidimensional Poverty Indicators by Household Gender.

The multidimensional poverty differences by household gender are presented in Table 2 and Figure 1. Excluding the asset size, housing and health dimensions, single-parent females exhibited higher levels than males across all other dimensions of multidimensional poverty. Specifically, in terms of the income dimension, the absolute poverty rate for females was 66.5%, compared to 60.8% for males, showing a statistically significant difference (χ^2^ = 10.440, *p* < 0.01). The rate for asset size, however, was higher among males (68.8%) than females (59.0%) (χ^2^ = 29.987, *p* < 0.001). Regarding the employment dimension, 22.0% of females were unemployed, compared to 16.1% of males, revealing a statistically significant difference (χ^2^ = 16.035, *p* < 0.001). In the social dimension, single-parent females and their children exhibited higher rates of experienced discrimination than their male counterparts (χ^2^ = 14.893, *p* < 0.001; χ^2^ = 7.969, *p* < 0.01).

### 3.3. Regression of Multidimensional Poverty and Depression

The results of the weighted regression analysis examining the association between multidimensional poverty and depression, stratified by household gender, are presented in Table 3. For male single-parent households, employment status (*b* = 1.94, 95% CI [0.93, 2.95]), health status (*b* = 3.34, 95% CI [2.41, 4.28]), and discrimination experienced as a single parent (*b* = 0.85, 95% CI [0.15, 1.56]) were significantly associated with depression. For female single-parent households, educational level, asset poverty, employment status, health status, and discrimination experienced as a single parent were significantly related to depression. Compared with those who had a middle-school education or less, high-school graduates reported significantly lower depression scores (*b* = −1.12, 95% CI [−2.08, −0.16]). Female single parents facing asset poverty (b = 0.58, 95% CI [0.30, 0.87]), unemployment (b = 0.64, 95% CI [0.18, 1.09]), poor health (b = 3.38, 95% CI [2.97, 3.78]), and discrimination as a single parent (b = 0.56, 95% CI [0.09, 1.03]) reported significantly higher depression scores. The overall models were statistically significant for both male (*F* = 15.19, adj *R*^2^ = 0.298) and female single-parent households (*F* = 24.72, adj *R*^2^ = 0.237).

To directly test whether the associations between multidimensional poverty and depression differ by household gender, a pooled regression model was estimated with sex entered as a dummy variable and interacted with each of the eight poverty indicators (Table 4). Across the sample as a whole, higher educational attainment (high school vs. middle school or less: *b* = −0.92, 95% CI [−1.71, −0.13]), employment status (*b* = 1.89, 95% CI [0.85, 2.92]), health status (*b* = 3.39, 95% CI [2.40, 4.37]), and discrimination experienced as a single parent (*b* = 0.89, 95% CI [0.20, 1.58]) were significantly associated with depression. Among the eight gender-interaction terms, only the interaction between employment status and gender was statistically significant (*b* = −1.22, 95% CI [−2.35, −0.10]), indicating that the association between employment-related poverty and depression was significantly stronger for male single parents than for female single parents. The interaction for absolute poverty approached significance (*b* = 0.54, 95% CI [−0.09, 1.16]). A joint Wald test of all eight gender-interaction terms indicated that, taken together, the associations between multidimensional poverty and depression did not differ significantly by gender (F = 1.55, *p* = 0.134).

### 3.4. Regression of Cumulative Multidimensional Poverty and Depression

**Figure 2:**
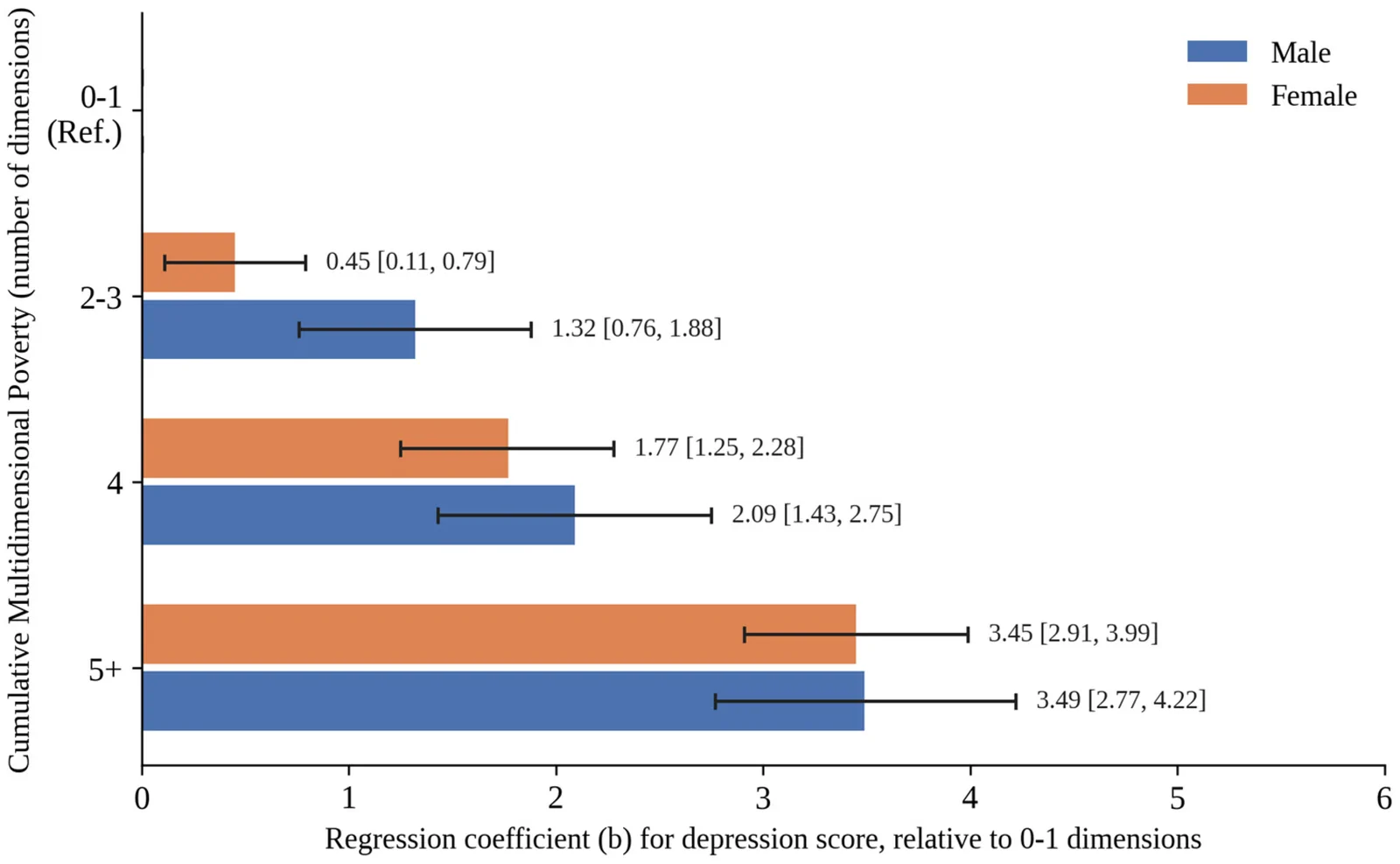
Depression Score by Cumulative Multidimensional Poverty and Gender.

Table 5 presents the results of the weighted regression analysis examining the association between cumulative multidimensional poverty and depression by household gender. For male single-parent households, depression scores increased significantly and monotonically with the number of poverty dimensions experienced. Compared with the reference group (0–1 dimensions), male single parents with 2–3 dimensions of poverty reported depression scores 1.32 points higher (95% CI [0.76, 1.88]), those with 4 dimensions reported scores 2.09 points higher (95% CI [1.43, 2.75]), and those with 5 or more dimensions reported scores 3.49 points higher (95% CI [2.77, 4.22]). No sociodemographic covariates reached statistical significance for male single parents in this model. For female single-parent households, a similar dose–response pattern emerged, alongside significant educational effects. Compared with those who had a middle-school education or less, female single parents with a high-school education (*b* = −1.44, 95% CI [−2.52, −0.35]), an associate degree (*b* = −1.33, 95% CI [−2.44, −0.23]), and a bachelor’s degree or higher (*b* = −1.26, 95% CI [−2.38, −0.14]) reported significantly lower depression scores. Relative to the 0–1 dimension reference group, female single parents with 2–3 dimensions of poverty reported depression scores 0.45 points higher (95% CI [0.11, 0.79]), those with 4 dimensions reported scores 1.77 points higher (95% CI [1.25, 2.28]), and those with 5 or more dimensions reported scores 3.45 points higher (95% CI [2.91, 3.99]). The overall models were statistically significant for both male (*F* = 13.78, adj *R*^2^ = 0.145) and female single-parent households (*F* = 18.62, adj *R*^2^ = 0.118) (Figure 2).

To examine whether the association between cumulative multidimensional poverty and depression differed by household gender, a pooled regression model was estimated with gender entered as a dummy variable interacting with the categorical cumulative poverty variable (Table 6). In the overall sample, higher educational attainment was significantly associated with lower depression (high school: *b* = −1.24, 95% CI [−2.13, −0.34]; bachelor’s degree or higher: *b* = −1.03, 95% CI [−1.95, −0.11]; associate degree: *b* = −0.94, 95% CI [−1.89, 0.00]), and depression scores rose significantly with the number of poverty dimensions experienced—2–3 dimensions (*b* = 1.32, 95% CI [0.75, 1.90]), 4 dimensions (*b* = 2.10, 95% CI [1.44, 2.76]), and 5 or more dimensions (*b* = 3.50, 95% CI [2.77, 4.22])—relative to the 0–1 reference group. Female single parents also reported significantly higher depression scores than male single parents overall (*b* = 0.59, 95% CI [0.22, 0.95]). Among the three gender-interaction terms, only the interaction between the 2–3 dimensions category and gender was statistically significant (*b* = −0.87, 95% CI [−1.54, −0.19]), indicating that the depression-elevating effect of moderate poverty accumulation (2–3 dimensions) was significantly stronger for male than for female single parents. The interactions for the 4-dimension category (*b* = −0.34, 95% CI [−1.18, 0.49]) and the 5-or-more category (*b* = −0.04, 95% CI [−0.93, 0.85]) were not statistically significant, suggesting that the gender gap observed at moderate levels of poverty accumulation narrowed and effectively disappeared at higher levels. A joint Wald test of all three gender-interaction terms indicated that, taken together, the association between cumulative multidimensional poverty and depression did not differ significantly by gender at the conventional 0.05 threshold, although the result approached significance (*F* = 2.24, *p* = 0.082).

## 4. Discussion

This study investigates the relationship between multidimensional poverty and depression among single-parent families and directly tests whether this relationship differs by household gender. To measure multidimensional poverty, five domains—income, employment, housing, health, and social dimensions—were analyzed. This research examines not only the individual effects of poverty within each dimension but also the cumulative impact of multidimensional poverty on depression, and formally tests for gender differences in both sets of associations. The key findings and implications of this study are as follows.

### 4.1. Multidimensional Poverty Prevalence Among Single-Parent Families

The findings of this study indicate that multidimensional poverty among single-parent families is most prevalent in the order of income, housing, health, social relations, and employment. Female-headed households exhibited higher poverty rates than their male counterparts across most domains, with a notable exception: asset poverty and housing area were more prevalent among male-headed households. As of 2024, 53.8% of single-parent families reported currently receiving basic livelihood security support, an increase from 45.8% in 2021 and 32.8% in 2018 [2], indicating that the foundation for economic self-reliance among single-parent families remains extremely fragile and that this dependence has grown steadily rather than being a temporary condition. Therefore, priority must be given to multifaceted economic interventions, such as asset-building support programs, which are closely linked to mitigating housing poverty in addition to direct income maintenance. Furthermore, to alleviate poverty in the social dimension, it is imperative to move beyond simple awareness campaigns. Practical programs must be implemented in tandem to eliminate social stigma toward single-parent families and to help them establish robust support networks within their local communities.

### 4.2. Influencing Factors of Multidimensional Poverty on Depression

This study analyzed the impact of multidimensional poverty on depression among single-parent families, specifically identifying gender-specific predictors. The results indicate that employment status, health levels, and perceived discrimination are common significant factors influencing depression. This suggests that mental health in single-parent households is determined not only by economic deprivation but also by physical functional capacity and social receptivity. The significance of employment and health as key predictors underscores that support policies must move beyond simple cash transfers. Employment instability leads to social isolation and diminished self-esteem, while poor health creates a vicious cycle where the inability to balance childcare and economic activity exacerbates depressive symptoms. Furthermore, the strong influence of ‘perceived discrimination’ confirms that the psychological distress of single parents stems from external environments—namely social stigma and prejudice—rather than internal deficits. Notably, for female single parents, educational attainment was identified as an additional critical variable, whereas household size was not significantly associated with depression in either gender in the present analysis. Consequently, policies for female-headed households should integrate expanded educational opportunities with the establishment of community-based care networks that support single parenting more broadly, regardless of household composition. In conclusion, addressing depression in single-parent families requires a comprehensive intervention strategy targeting multidimensional poverty. A holistic improvement in their quality of life can only be realized through integrated efforts that combine economic self-reliance programs, health promotion, and institutional mechanisms designed to dismantle social discrimination.

### 4.3. Association Between Cumulative Multidimensional Poverty on Depression

This study demonstrates that an increase in experienced multidimensional poverty correlates with a higher likelihood of depression for both male and female single parents. A formal test of the gender-by-poverty interaction indicated that this dose–response pattern did not differ significantly by gender overall, although the effect of moderate poverty accumulation (2–3 dimensions) was significantly stronger for male single parents, with this gender gap narrowing at higher levels of cumulative poverty. While previous research has often focused on the independent effects of isolated poverty domains—a method criticized for potentially overestimating the influence of specific factors [25]—this study emphasizes the cumulative effect of poverty. Given that various dimensions of poverty are interrelated and tend to occur concurrently [26], confirming their aggregate impact provides a more robust understanding of the mechanisms driving depression. Although the depression–poverty gradient itself was not more pronounced for female single parents, the demographic weight of this vulnerability remains disproportionately borne by women: as of 2024, the proportion of female householders within single-parent families stood at 66.3%, more than double the national average of 32.7% for all households as of 2020 [27]. This disparity aligns with the concept of ‘feminization of poverty,’ where gender and marital status intersect to create a dual vulnerability that increases the risk of multidimensional poverty. Consequently, policy initiatives should prioritize not only basic income security for low-income female householders but also the provision of substantive educational opportunities and vocational retraining to establish a foundation for structural poverty alleviation, while employment-focused interventions should be prioritized for male single parents given their distinct vulnerability to employment-related poverty.

### 4.4. Implications

Based on the finding that cumulative multidimensional poverty reveals a dose-dependent association with depression that domain-specific analysis alone does not capture, this study offers the following implications for policy and practice. First, integrated case management should be implemented. The evidence of the cumulative effect of poverty underscores the limitations of fragmented support systems. It is imperative to transition toward a ‘One-stop Integrated Case Management’ system that consolidates services currently dispersed across income, housing, employment, and health sectors. Such a system would facilitate the early identification and holistic support of high-risk single-parent households facing multiple deprivations simultaneously. Second, gender-sensitive interventions should target the dual vulnerability facing female single parents. To address the ‘feminization of poverty,’ interventions must be tailored to the specific needs of female single parents. Given that female single parents face significantly higher poverty in the income, employment, and social dimensions, and that lower educational attainment correlates significantly with higher depression among this group, policy focus should shift toward providing substantive re-education and vocational training to foster upward labor market mobility. Furthermore, to mitigate the psychological isolation and caregiving burden of solo parenting, the development of community-based care networks is essential. At the same time, employment-support interventions should be prioritized for male single parents, given the significantly stronger association between employment-related poverty and depression observed in this group. Third, dismantling social stigma and strengthening psychosocial support are equally critical. The strong predictive power of ‘perceived discrimination’ on depression indicates that social stigma is a critical determinant of mental health. Nationwide campaigns are required to reshape public perception and decouple the automatic association between single parenthood and poverty. Additionally, institutionalizing comprehensive counseling programs that link health promotion with employment assistance—alongside fostering peer support groups—will be vital in enhancing the psychosocial resilience of single-parent families.

### 4.5. Limitations

Despite the significant findings regarding the gender-specific impacts of multidimensional poverty on depression, this study has several limitations that should be addressed in future research. First, this research is a cross-sectional study based on data from a specific point in time, which limits the ability to establish a definitive causal relationship between multidimensional poverty and depression. While poverty may lead to depression, the reverse—where depressive symptoms hinder economic activity and exacerbate poverty—cannot be ruled out. Future research utilizing longitudinal panel data is necessary to track the dynamic trajectories of how cumulative poverty impacts mental health over time. Second, there are constraints related to the measurement indicators of multidimensional poverty. This study relied primarily on the responses of household heads to assess various domains. To reflect actual living standards more accurately, future studies should develop more sophisticated measurement tools that integrate objective socioeconomic indicators with subjective measures of social deprivation. Third, the study did not fully account for the heterogeneity within single-parent families. The underlying causes of single parenthood (e.g., divorce, bereavement, or never-married status) and the developmental stages of children may significantly influence poverty patterns and psychological distress. While this study focused on gender disparities, subsequent research should conduct disaggregated analyses that consider the diverse characteristics of various subgroups within the single-parent population. Fourth, the discrimination indicators relied on respondents’ subjective perception and self-classification of an experience as discriminatory, which may vary across individuals; the relatively high affirmative rate should therefore be interpreted with this in mind rather than as an objective incidence rate. In particular, discrimination experienced by the child was measured through parental report rather than the child’s own account, and parents—especially those of older children—may not have full knowledge of such experiences. This proxy measurement may attenuate the true association between child-directed discrimination and the outcomes examined in this study.

## 5. Conclusions

This study aimed to investigate the structure of multidimensional poverty among single-parent families and directly test whether its effects on depression differ by gender. The findings confirm that single-parent households experience severe deprivation across multiple domains—including income, housing, and health—and that these factors exert a cumulative, rather than merely independent, influence on psychological distress. Notably, female single parents exhibited higher poverty rates in the income, employment, and social dimensions compared to their male counterparts, while male single parents showed higher asset poverty. Educational attainment emerged as a significant predictor of depression specific to female single parents, whereas employment-related poverty carried a significantly greater mental health burden for male single parents. These results underscore how structural issues, namely the ‘feminization of poverty’ and ‘dual vulnerability,’ manifest within the single-parent population, with male and female single parents facing distinct rather than uniformly gender-skewed configurations of risk. In conclusion, this research suggests that the policy paradigm for single-parent families must shift beyond simple financial subsidies toward a multidimensional and integrated approach that encompasses housing stability, employment assistance, health promotion, and the eradication of social stigma. It is hoped that the evidence provided by this study will serve as a foundational resource for developing gender-sensitive policies aimed at enhancing the psychosocial well-being of and substantially improving the quality of life for single-parent families.

## Figures and Tables

**Table 1:** The demographic characteristics of Respondents.

Variables		Total (N = 3315)	Male (N = 1118)	Female (N = 2197)	*χ*^2^/*t*
Age	Under 40	765 (23.1)	154 (13.8)	611 (27.8)	196.976 ***
	40s	1875 (56.6)	596 (53.3)	1279 (58.2)	
	Over 50	675 (20.4)	368 (32.9)	307 (14.0)	
Education Level	Middle school or lower	121 (3.7)	46 (4.1)	75 (3.4)	21.803 ***
	High school	1830 (55.2)	649 (58.1)	1181 (53.8)	
	Associate degree	669 (20.2)	175 (15.7)	494 (22.5)	
	Bachelor’s degree or higher	695 (21.0)	248 (22.2)	447 (20.3)	
Household Size	Two	1505 (45.4)	455 (40.7)	1050 (47.8)	17.549 **
	Three	1230 (37.1)	436 (39.0)	794 (36.1)	
	Four	420 (12.7)	166 (14.8)	254 (11.6)	
	Five or more	160 (4.8)	61 (5.5)	99 (4.5)	
Duration of single parenthood	Less than 5 years	1339 (40.4)	396 (35.4)	943 (42.9)	17.522 ***
	5–10 years	1364 (41.1)	494 (44.2)	870 (39.6)	
	More than 10 years	612 (18.5)	228 (20.4)	384 (17.5)	
Youngest child	Pre-school	361 (10.9)	89 (8.0)	272 (12.4)	20.557 ***
	Elementary school	1255 (37.9)	407 (36.4)	848 (38.6)	
	Middle school and above	1699 (51.3)	622 (55.6)	1077 (49.0)	
Depression (M ± SD)		2.90 ± 3.96	2.66 ± 3.74	3.03 ± 4.06	−2.61 **

** *p* < 0.01, *** *p* < 0.001.

**Table 2:** Multidimensional Poverty Differences by Household Gender.

Variables		Total (N = 3315)	Male (N = 1118)	Female (N = 2197)	*χ* ^2^
Income	Absolute poverty	2141 (64.6)	680 (60.8)	1461 (66.5)	10.440 **
	Asset size	2066 (62.3)	769 (68.8)	1297 (59.0)	29.987 ***
Employment	Employment status	663 (20.0)	180 (16.1)	483 (22.0)	16.035 ***
Housing	Housing area	844 (25.5)	294 (26.3)	550 (25.0)	0.623
	Housing type	1957 (59.0)	656 (58.7)	1301 (59.2)	0.090
Health	Health status	1088 (32.8)	365 (32.6)	723 (32.9)	0.023
Social	Discrimination to Single-parent	885 (26.7)	252 (22.5)	633 (28.8)	14.893 ***
	Discrimination to Child	704 (21.2)	206 (18.4)	498 (22.7)	7.969 **

** *p* < 0.01, *** *p* < 0.001.

**Table 3:** Regression of Multidimensional Poverty and Depression by Household Gender.

Variables		Male			Female		
		b (SE)	95% CI	t	b(SE)	95% CI	t
Age		0.00 (0.02)	[−0.04, 0.05]	0.13	−0.01 (0.01)	[−0.03, 0.02]	−0.41
Education	Middle school (Ref.)						
	High school	−0.19 (0.62)	[−1.40, 1.02]	−0.30	−1.12 (0.49)	[−2.08, −0.16]	−2.29 *
	Associate degree	0.53 (0.69)	[−0.82, 1.89]	0.77	−0.93 (0.50)	[−1.92, 0.05]	−1.85
	Bachelor’s degree or higher	0.34 (0.63)	[−0.90, 1.57]	0.53	−0.99 (0.51)	[−1.99, 0.01]	−1.93
Household Size		0.00 (0.10)	[−0.20, 0.19]	−0.01	−0.07 (0.09)	[−0.25, 0.11]	−0.79
Duration of single parenthood	Less than 5 years (Ref.)						
	5–10 years	0.29 (0.22)	[−0.14, 0.73]	1.33	−0.01 (0.17)	[−0.34, 0.33]	−0.05
	More than 10 years	0.42 (0.41)	[−0.39, 1.23]	1.02	−0.03 (0.24)	[−0.51, 0.44]	−0.14
Youngest child	Pre-school (Ref.)						
	Elementary School	0.55 (0.30)	[−0.04, 1.13]	1.82	0.17 (0.26)	[−0.34, 0.69]	0.66
	Middle School and Above	0.56 (0.38)	[−0.19, 1.31]	1.47	0.16 (0.31)	[−0.44, 0.75]	0.51
Income	Absolute poverty	−0.17 (0.27)	[−0.71, 0.37]	−0.62	0.31 (0.16)	[0.00, 0.62]	1.95
	Asset size	0.37 (0.22)	[−0.07, 0.81]	1.65	0.58 (0.15)	[0.30, 0.87]	3.99 ***
Employment	Employment status	1.94 (0.52)	[0.93, 2.95]	3.77 ***	0.64 (0.23)	[0.18, 1.09]	2.76 **
Housing	Housing area	0.04 (0.27)	[−0.48, 0.57]	0.17	−0.03 (0.19)	[−0.40, 0.34]	−0.18
	Housing type	0.10 (0.22)	[−0.33, 0.52]	0.45	−0.14 (0.15)	[−0.45, 0.16]	−0.94
Health	Health status	3.34 (0.48)	[2.41, 4.28]	7.00 ***	3.38 (0.21)	[2.97, 3.78]	16.40 ***
Social	Discrimination to Single-parent	0.85 (0.36)	[0.15, 1.56]	2.38 *	0.56 (0.24)	[0.09, 1.03]	2.32 *
	Discrimination to Child	−0.19 (0.37)	[−0.91, 0.53]	−0.52	0.39 (0.28)	[−0.16, 0.94]	1.40
Intercept		0.01 (1.10)	[−2.15, 2.17]	0.01	2.32 (0.83)	[0.70, 3.93]	2.80 **
F		15.19 ***			24.72 ***		
R^2^		0.308			0.243		
adj R^2^		0.298			0.237		

* *p* < 0.05, ** *p* < 0.01, *** *p* < 0.001.

**Table 4:** Regression of Multidimensional Poverty and Depression with gender interaction.

Variables		Main Effect			Gender Interaction		
		b (SE)	95% CI	t	b(SE)	95% CI	t
Age		−0.00 (0.01)	[−0.03, 0.02]	−0.35	—	—	—
Education	Middle school (Ref.)						
	High school	−0.92 (0.40)	[−1.71, −0.13]	−2.27 *			
	Associate degree	−0.60 (0.42)	[−1.43, 0.23]	−1.41			
	Bachelor’s degree or higher	−0.67 (0.42)	[−1.49, 0.15]	−1.59			
Household Size		−0.05 (0.07)	[−0.19, 0.09]	−0.68			
Duration of single parenthood	Less than 5 years (Ref.)						
	5–10 years	0.08 (0.14)	[−0.19, 0.34]	0.55			
	More than 10 years	0.09 (0.21)	[−0.32, 0.51]	0.44			
Youngest child	Pre-school (Ref.)						
	Elementary School	0.24 (0.21)	[−0.17, 0.66]	1.15			
	Middle School and Above	0.25 (0.25)	[−0.24, 0.74]	0.99			
Gender (Ref. male)		−0.04 (0.22)	[−0.48, 0.40]	−0.18			
Income	Absolute poverty	−0.22 (0.28)	[−0.77, 0.32]	−0.80	0.54 (0.32)	[−0.09, 1.16]	1.69
	Asset size	0.38 (0.23)	[−0.07, 0.82]	1.65	0.22 (0.27)	[−0.31, 0.75]	0.80
Employment	Employment status	1.89 (0.53)	[0.85, 2.92]	3.58 ***	−1.22 (0.57)	[−2.35, −0.10]	−2.13 *
Housing	Housing area	−0.03 (0.27)	[−0.55, 0.50]	−0.10	0.00 (0.33)	[−0.63, 0.64]	0.01
	Housing type	0.07 (0.22)	[−0.37, 0.50]	0.31	−0.20 (0.27)	[−0.73, 0.32]	−0.76
Health	Health status	3.39 (0.50)	[2.40, 4.37]	6.75 ***	−0.02 (0.54)	[−1.07, 1.04]	−0.03
Social	Discrimination to Single-parent	0.89 (0.35)	[0.20, 1.58]	2.52 *	−0.33 (0.43)	[−1.17, 0.50]	−0.78
	Discrimination to Child	−0.19 (0.37)	[−0.91, 0.52]	−0.53	0.58 (0.46)	[−0.32, 1.48]	1.26
Intercept		1.82 (0.70)	[0.44, 3.20]	2.59 *			
F			24.96 ***				
R^2^			0.262				
adj R^2^			0.257				
Joint test of all interactions			F(8, 3307) = 1.55, *p* = 0.134				

Note: Results are from a pooled model; gender coded as a dummy (male = ref). “Gender Interaction” = how the effect differs for females; * *p* < 0.05, *** *p* < 0.001.

**Table 5:** Regression of Cumulative Multidimensional Poverty and Depression by Household Gender.

Variables		Male			Female		
		b (SE)	95% CI	t	b(SE)	95% CI	t
Age		0.04 (0.03)	[−0.01, 0.08]	1.41	0.01 (0.02)	[−0.02, 0.04]	0.78
Education Level	Middle school (Ref.)						
	High school	−0.53 (0.69)	[−1.87, 0.82]	−0.77	−1.44 (0.55)	[−2.52, −0.35]	−2.60 **
	Associate degree	0.35 (0.80)	[−1.22, 1.91]	0.43	−1.33 (0.56)	[−2.44, −0.23]	−2.37 *
	Bachelor’s degree or higher	−0.16 (0.69)	[−1.51, 1.19]	−0.23	−1.26 (0.57)	[−2.38, −0.14]	−2.21 *
Household Size		−0.02 (0.11)	[−0.23, 0.20]	−0.16	−0.07 (0.10)	[−0.26, 0.12]	−0.71
Duration of single parenthood	Less than 5 years (Ref.)						
	5–10 years	0.47 (0.25)	[−0.02, 0.97]	1.87	0.04 (0.19)	[−0.32, 0.41]	0.24
	More than 10 years	0.90 (0.53)	[−0.14, 1.94]	1.69	0.14 (0.26)	[−0.37, 0.66]	0.54
Youngest child	Pre-school (Ref.)						
	Elementary School	0.50 (0.30)	[−0.09, 1.10]	1.65	0.19 (0.27)	[−0.34, 0.72]	0.70
	Middle School and Above	0.41 (0.40)	[−0.37, 1.19]	1.03	0.23 (0.33)	[−0.41, 0.87]	0.71
Cumulative Multidimensional Poverty	0–1 (Ref.)						
	2–3	1.32 (0.28)	[0.76, 1.88]	4.64 ***	0.45 (0.17)	[0.11, 0.79]	2.61 **
	4	2.09 (0.34)	[1.43, 2.75]	6.22 ***	1.77 (0.26)	[1.25, 2.28]	6.75 ***
	More than 5	3.49 (0.37)	[2.77, 4.22]	9.41 ***	3.45 (0.27)	[2.91, 3.99]	12.63 ***
Intercept		−1.01 (1.24)	[−3.45, 1.42]	−0.82	2.40 (0.87)	[0.70, 4.10]	2.76 **
F		13.78 ***			18.62 ***		
R^2^		0.154			0.123		
adj R^2^		0.145			0.118		

* *p* < 0.05, ** *p* < 0.01, *** *p* < 0.001.

**Table 6:** Regression of Cumulative Multidimensional Poverty and Depression by Household Gender.

Variables		Main Effect			Gender Interaction		
		b (SE)	95% CI	t	b(SE)	95% CI	t
Age		0.02 (0.01)	[−0.01, 0.04]	1.34			
Education Level	Middle school (Ref.)						
	High school	−1.24 (0.46)	[−2.13, −0.34]	−2.71 **			
	Associate degree	−0.94 (0.48)	[−1.89, 0.00]	−1.95			
	Bachelor’s degree or higher	−1.03 (0.47)	[−1.95, −0.11]	−2.19 *			
Household Size		−0.05 (0.08)	[−0.20, 0.10]	−0.64			
Duration of single parenthood	Less than 5 years (Ref.)						
	5–10 years	0.16 (0.15)	[−0.13, 0.46]	1.09			
	More than 10 years	0.35 (0.24)	[−0.13, 0.83]	1.45			
Youngest child	Pre-school (Ref.)						
	Elementary School	0.24 (0.22)	[−0.19, 0.67]	1.08			
	Middle School and Above	0.24 (0.27)	[−0.28, 0.76]	0.92			
Gender (Ref. male)		0.59 (0.19)	[0.22, 0.95]	3.14 **			
Cumulative Multidimensional Poverty	0–1 (Ref.)						
	2–3	1.32 (0.30)	[0.75, 1.90]	4.48 ***	−0.87 (0.34)	[−1.54, −0.19]	−2.53 *
	4	2.10 (0.34)	[1.44, 2.76]	6.25 ***	−0.34 (0.42)	[−1.18, 0.49]	−0.81
	More than 5	3.50 (0.37)	[2.77, 4.22]	9.41 ***	−0.04 (0.45)	[−0.93, 0.85]	−0.09
Intercept		1.14 (0.75)	[−0.34, 2.62]	1.51			
F			24.59 ***				
R^2^			0.133				
adj R^2^			0.128				
Joint test of interaction			F(3, 3312) = 2.24, *p* = 0.082				

* *p* < 0.05, ** *p* < 0.01, *** *p* < 0.001.

## Data Availability

The data presented in this study are openly available from the Ministry of Gender Equality and Family at https://www.data.go.kr/data/15114757/fileData.do (accessed on 30 June 2026).
